# How Are Substrate Binding and Catalysis Affected by Mutating Glu_127_ and Arg_161_ in Prolyl-4-hydroxylase? A QM/MM and MD Study

**DOI:** 10.3389/fchem.2017.00094

**Published:** 2017-11-09

**Authors:** Amy Timmins, Sam P. de Visser

**Affiliations:** School of Chemical Engineering and Analytical Science, Manchester Institute of Biotechnology, The University of Manchester, Manchester, United Kingdom

**Keywords:** quantum mechanics/molecular mechanics, enzyme mechanism, enzyme catalysis, mutations, density functional theory

## Abstract

Prolyl-4-hydroxylase is a vital enzyme for human physiology involved in the biosynthesis of 4-hydroxyproline, an essential component for collagen formation. The enzyme performs a unique stereo- and regioselective hydroxylation at the C^4^ position of proline despite the fact that the C^5^ hydrogen atoms should be thermodynamically easier to abstract. To gain insight into the mechanism and find the origin of this regioselectivity, we have done a quantum mechanics/molecular mechanics (QM/MM) study on wildtype and mutant structures. In a previous study (Timmins et al., 2017) we identified several active site residues critical for substrate binding and positioning. In particular, the Glu_127_ and Arg_161_ were shown to form multiple hydrogen bonding and ion-dipole interactions with substrate and could thereby affect the regio- and stereoselectivity of the reaction. In this work, we decided to test that hypothesis and report a QM/MM and molecular dynamics (MD) study on prolyl-4-hydroxylase and several active site mutants where Glu_127_ or Arg_161_ are mutated for Asp, Gln, or Lys. Thus, the R161D and R161Q mutants give very high barriers for hydrogen atom abstraction from any proline C–H bond and therefore will be inactive. The R161K mutant, by contrast, sees the regio- and stereoselectivity of the reaction change but still is expected to hydroxylate proline at room temperature. By contrast, the Glu_127_ mutants E127D and E127Q show possible changes in regioselectivity with the former being more probable to react compared to the latter.

## Introduction

Metalloenzymes play vital roles in nature and are involved in biosynthesis as well as biodegradation of compounds (Solomon et al., 2000; Costas et al., 2004; Abu-Omar et al., 2005; Kryatov et al., 2005; Bruijnincx et al., 2008; Kadish et al., 2010). Due to its large natural abundance often metalloenzymes contain one or more iron centers; however, in this work we will restrict ourselves to mononuclear iron enzymes only and particularly those that utilize molecular oxygen. In general, iron containing dioxygenases and monoxygenases use one molecule of molecular oxygen in their catalytic cycle and either transfer both oxygen atoms to substrate(s) or a single one with a water molecule as by-product (Sub = substrate), Equations 1, 2.

(1) $$\begin{array}{r} \left(\text{nonheme}\right)\text{F}{\text{e}}^{\text{III}}+\text{Sub}+{\text{O}}_{2}\to \left(\text{nonheme}\right)\text{F}{\text{e}}^{\text{III}}+\text{Sub}{\text{O}}_{2} \end{array}$$

(2) $$\begin{array}{r} \left(\text{heme}\right)\text{F}{\text{e}}^{\text{III}}+\text{Sub}+{\text{O}}_{2}+2{\text{H}}^{+}+2{\text{e}}^{-}\to \left(\text{heme}\right)\text{F}{\text{e}}^{\text{III}}+\text{SubO}+{\text{H}}_{2}\text{O} \end{array}$$

Thus, heme monoxygenases, like the cytochromes P450, react as monoxygenases and proceed through a catalytic cycle starting from an iron(III)-heme resting state with a protein cysteinate and a water molecule in the fifth and sixth iron ligand positions, respectively (Meunier et al., 2004; Ortiz de Montellano, 2004, 2010; Denisov et al., 2005; Kadish et al., 2010). The water molecule is released after substrate binding, which triggers a spin state change from low-spin to high-spin and enables molecular oxygen binding to the iron center. The iron-superoxo is subsequently reduced and protonated to form an iron(III)-hydroperoxo(heme) complex, also called Compound 0 (Meunier et al., 2004; Denisov et al., 2005; Shaik et al., 2005; Ortiz de Montellano, 2010). A final protonation step gives water and an iron(IV)-oxo(heme cation radical) species called Compound I (CpdI) (de Visser et al., 2003; Rittle and Green, 2010; de Visser and Kumar, 2011). Now, CpdI is the active species of P450 enzymes and reacts with substrates through oxygen atom transfer and hence converts aliphatic groups to alcohols (Ogliaro et al., 2000b; de Visser et al., 2004; Ji et al., 2015), C = C double bonds to epoxides (de Visser et al., 2001, 2002b; Sainna et al., 2015), sulfides to sulfoxides (Sharma et al., 2003; Kumar et al., 2005b, 2011a), and arenes to phenols (de Visser and Shaik, 2003; de Visser, 2006c; Cantú Reinhard et al., 2016b). The mechanism of these reactions has been established with computational modeling including density functional theory (de Visser, 2012; Blomberg et al., 2014). In recent years full calculations on enzymatic structures were done and identified the effect of the protein, substrate orientation and hydrogen bonding interactions on the kinetics and thermodynamics of the reaction and the product distributions of P450 catalyzed reaction mechanisms. Thus, it was shown with small model complexes that hydrogen bond donations toward the axial thiolate ligand affected the electron affinity of this cysteinate residue, which led to a push-effect of electrons to the heme that influenced its redox potential and hence catalytic potential (Ogliaro et al., 2000a; de Visser et al., 2002a; Schöneboom et al., 2002). In the heme enzyme cytochrome *c* peroxidase, a model that included a cation binding site reproduced the experimentally characterized electronic configuration, and highlighted the importance of long-range electrostatic effects in enzyme models (de Visser, 2005). Because of these long-range effects, more and more computational studies are done using full enzymatic systems using the Quantum Mechanics/Molecular Mechanics (QM/MM) technique.

**Figure 1:**
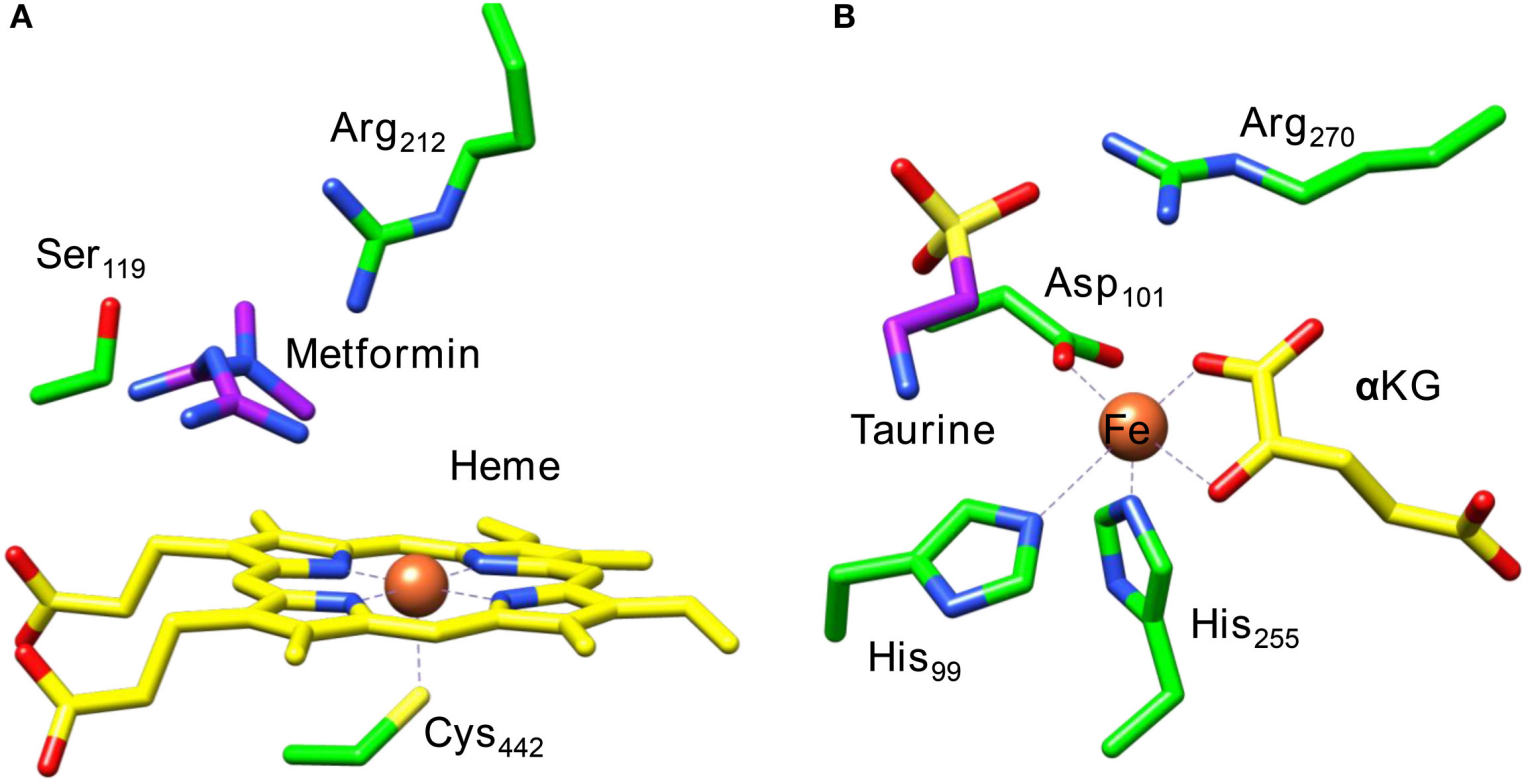
Extracts from the crystal structure coordinates of cytochrome P450 **(A)** and taurine/α-ketoglutarate dioxygenase **(B)** as taken from the 5G5J and 1OS7 pdb files.

Figure 1 displays the active site structures of (Figure 1A) cytochrome P450 and (Figure 1B) taurine/α-ketoglutarate dioxygenase (TauD) as a structural comparison (O'Brien et al., 2003; Guo and Sevrioukova, 2017). Thus, the P450s are heme enzymes, where the heme is linked to the protein backbone through an interaction of the metal with a cysteinate residue (the axial ligand). On the distal site of the heme the substrate binds, which is the drug molecule metformin in the 5G5J protein databank (pdb) file. The distal site of the heme has several hydrogen bonding and polar residues, such as Ser_119_ and Arg_212_, the former has been proposed to be involved in the proton relay mechanisms during the catalytic cycle (Kumar et al., 2005a), whereas the latter holds the substrate through a salt bridge into position.

A second iron enzyme class that utilizes molecular oxygen is the nonheme iron dioxygenases (Bugg, 2001; Ryle and Hausinger, 2002; Solomon et al., 2013). These dioxygenases are found in all forms of life and are involved in the biosynthesis of antibiotics (Choroba et al., 2000; Higgins et al., 2005; Siitonen et al., 2016), DNA and RNA repair enzymes (O'Brien, 2006; Yi et al., 2009), as well as the metabolism of toxic natural compounds such as cysteine (Stipanuk, 2004; Straganz and Nidetzky, 2006; de Visser, 2009; Buongiorno and Straganz, 2013). These enzymes are structurally very different from the heme monoxygenases as they link the iron atom to the protein with only amino acid side chains such as His, Asp, or Glu residues. Usually, the nonheme iron dioxygenases contain a facial triad of amino acid ligands with two histidine and one carboxylate group, i.e., 2-His/1-Asp, Glu (Que, 2000; Kovaleva and Lipscomb, 2008). As an example of a dioxygenase with these ligand features we show in Figure 1B the active site of taurine/α-ketoglutarate dioxygenase (TauD). TauD is a dioxygenase involved in the metabolism of cysteine, whereby it converts taurine to hydroxy-taurine. The 1OS7 pdb file (O'Brien et al., 2003) is a substrate and α-ketoglutarate (α-KG) bound structure of TauD with the iron bound to the protein through the side chains of residues His_99_, Asp_101_ and His_255_. Substrate taurine is located nearby the metal and is held in position through a salt bridge with residue Arg_270_. Co-substrate α-KG is bound to the metal as a bidentate ligand through the keto and acid groups.

**Figure 2:**
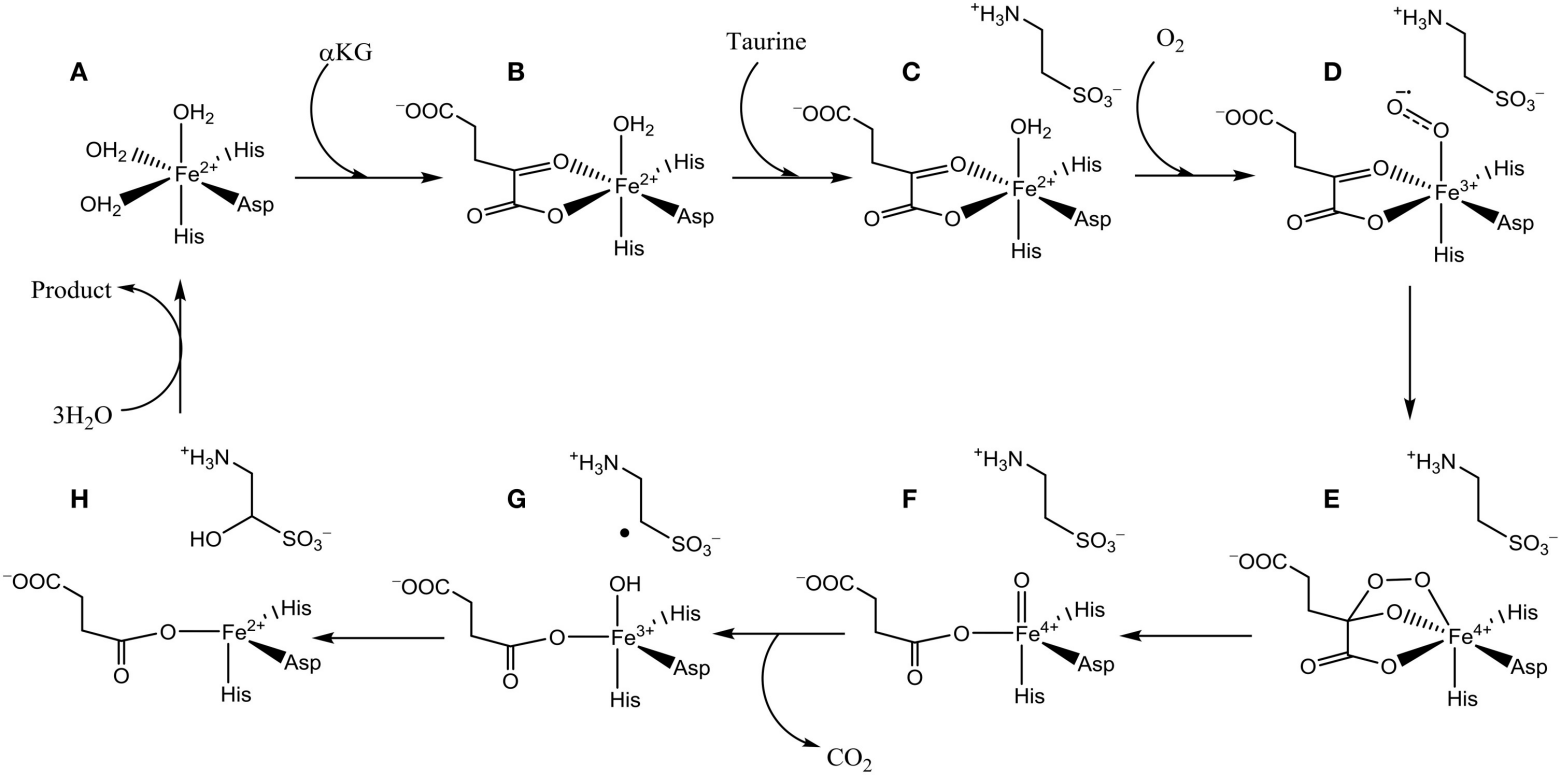
Catalytic cycle of nonheme iron dioxygenases. Structures **(A–H)** are defined in the text.

The catalytic cycle of TauD has been established through a combination of experimental and computational studies (Borowski et al., 2004; Bollinger et al., 2005; de Visser, 2006a,b, 2007; Godfrey et al., 2008). Figure 2 schematically depicts the catalytic cycle of TauD specifically and starts from the resting state structure where iron is bound to the 2-His/1-Asp ligand system and the other ligand positions of the metal are occupied by three water molecules (structure A). When α-KG enters the pocket two water molecules are displaced and replaced by the keto and acid groups of α-KG (structure B). In the next step, substrate taurine binds, which displaces the last water molecule from iron (structure C) and is replaced by molecular oxygen that binds as an iron(III)-superoxo (structure D). Subsequently, the superoxo group attacks the α-keto position of α-KG to form a bicyclic ring-structure (structure E). In the next step, the dioxygen bond breaks to form a peracid succinate with the release of CO_2_. Finally, the peracid bond breaks and splits into an iron(IV)-oxo species and succinate (structure F). Iron(IV)-oxo is known to be a powerful oxidant that abstracts a hydrogen atom from taurine to give an iron(III)-hydroxo group (structure G) and the hydroxyl radical is then rebound to form hydroxy-taurine as product (structure H). Products hydroxy-taurine and succinate are released from the iron center and their positions are replaced by water molecules to bring the catalytic cycle back into the resting state.

**Scheme 1:**
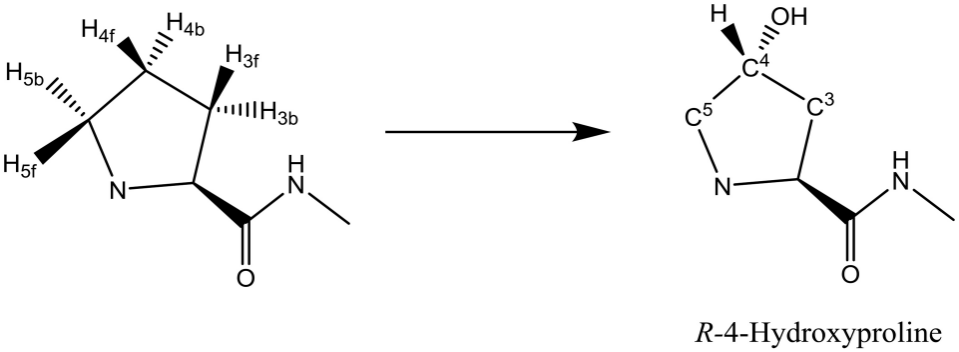
Conversion of a proline residue in a protein to R-4-hydroxyproline by P4H.

Another nonheme iron dioxygenase with a catalytic cycle similar to TauD is prolyl-4-hydroxylase (P4H), which regio- and stereospecifically hydroxylates a proline residue in a protein to *R*-4-hydroxyproline, Scheme 1. Product *R*-4-hydroxyproline is a common amino acid in animals and plants and has functions in collagen, where it enables crosslinking between individual strands. In addition, it is relevant to the synthesis of the hypoxia induced factor in animals (McDonough et al., 2006).

A range of biochemical and spectroscopic studies on P4H established key details of the catalytic cycle. Thus, reactions of P4H with (de Visser et al., 2001) ^18^O_2_ provided evidence of the transfer of one atom of molecular oxygen to proline (Myllyharju and Kivirikko, 1997). Low-temperature Mössbauer, electron paramagnetic resonance (EPR) and UV-Vis absorption spectroscopic studies characterized several intermediates in the catalytic cycle, including the iron(IV)-oxo species (Hoffart et al., 2006). It was shown that the iron(IV)-oxo species has a quintet spin ground state and reacts with the substrate through a rate-determining hydrogen atom abstraction. In particular, rate constants for the reaction with taurine and taurine-d_2_ gave a large kinetic isotope effect. To confirm the reaction mechanism computational studies on the catalytic cycle of P4H were performed: One study using an active site model complex (Karamzadeh et al., 2010) and another using the full enzyme structure with QM/MM (Timmins et al., 2017). These studies established the technical details of the catalytic cycle and confirmed the mechanism shown above in Figure 2. Furthermore, key functions of several amino acids were identified related to substrate positioning and product release as will be described in more detail later.

**Scheme 2:**
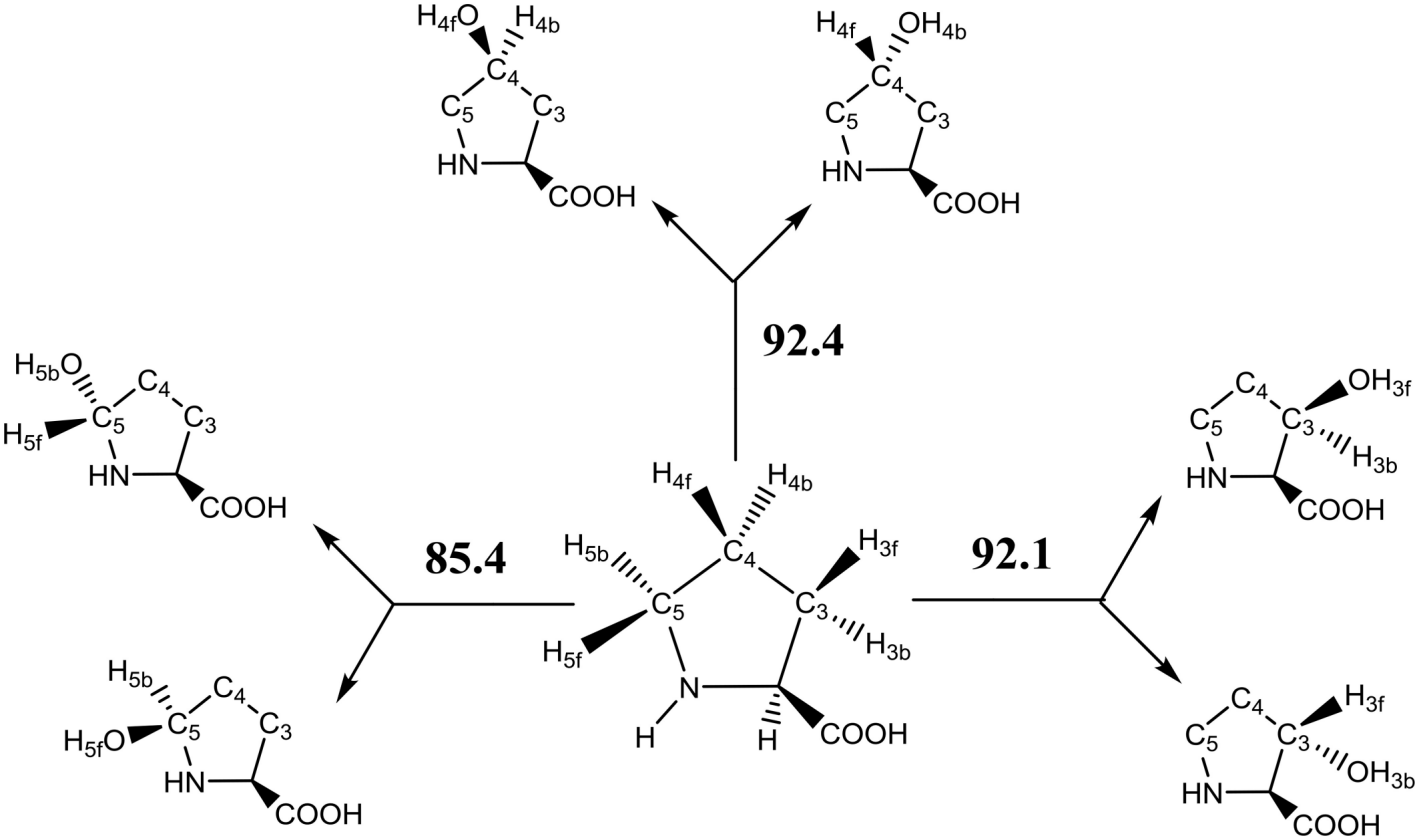
Aliphatic C–H bonds of proline and definition of atom labels and their bond dissociation free energies (BDFE in kcal mol−1).

The key step in the catalytic cycle of P4H is the hydrogen atom abstraction of substrate by the iron(IV)-oxo intermediate (Hoffart et al., 2006), which was shown to be rate-determining. In principle, substrate proline has six aliphatic C–H bonds at positions C^3^, C^4^, and C^5^ that could lead to six different product isomers, Scheme 2. We label the two hydrogen atoms on C^3^, C^4^, and C^5^ as front (f) or back (b). Also shown in Scheme 2 are bond dissociation free energies (BDFE) of each of these C–H bonds as calculated at UB3LYP/6-311+G^*^ as the difference in free energy of proline and the sum of a hydrogen atom and [proline – H^•^]. As can be seen, the C–H bond strength at the C^3^ and C^4^ positions in proline are comparable, while the one at the C^5^ position is much weaker in energy. Therefore, in the gas-phase proline hydroxylation should happen at the C^5^ position as it is the weakest bond to break rather than at the thermodynamically unfavorable C^4^ position. How P4H prevents hydroxylation of the weaker C^5^ position in favor of hydroxylation at the C^4^ position is the topic of this paper.

In addition to studies on wildtype (WT) P4H, we looked at the structure and catalytic properties of two active site mutants where the Glu_127_ and Arg_161_ residues were mutated to alternative groups. A previous study (Timmins et al., 2017) identified these two amino acids as key for substrate positioning in the substrate binding pocket and hence mutating them to a different amino acid should have a considerable effect.

## Methods

The calculations presented in this work follow previously described and benchmarked methods using QM/MM (Porro et al., 2009; Kumar et al., 2011b; Quesne et al., 2014, 2016a; Faponle et al., 2017; Li et al., 2017). Specifically, our previous work on the mechanism of the possible reaction channels of cytochrome P450 decarboxylase leading to decarboxylation of fatty acids or hydroxylation of fatty acids, predicted the correct regioselectivity of the reaction as compared to experiment and reproduced experimentally determined kinetic isotope effects (Faponle et al., 2016). Furthermore, QM/MM studies on 1-H-3-hydroxy-4-oxoquinaldine 2,4-dioxygenase focused on the rate-determining step of the co-factor independent reaction of substrate with molecular oxygen and predicted a rate constant in good agreement with experiment and explained how this enzyme functions without a metal cofactor present (Hernández-Ortega et al., 2014, 2015). Very recently, we used QM/MM modeling to predict spectroscopic fingerprints of short-lived catalytic cycle intermediates and used this on cysteine dioxygenase enzymes. Calculated UV-Vis absorption spectra and Mössbauer and EPR parameters enabled the experimental characterization of a short-lived oxygen-bound intermediate (Fellner et al., 2016; Tchesnokov et al., 2016).

### Model set-up

Our QM/MM starting point structures were set-up using previously described methods and procedures (Quesne et al., 2016a; Timmins et al., 2017), which we will summarize briefly here. The crystal structure coordinates from the 3GZE pdb file was used as a starting point for all models (Koski et al., 2009). The 3GZE pdb file represents a resting state P4H structure with Zn^2+^, pyridine-dicarboxylate co-substrate mimic and the (Ser-Pro)_5_ peptide chain bound. The active site zinc(II)-water(pyridine-dicarboxylate) was replaced with iron(IV)-oxo(succinate) manually with an Fe–O distance of 1.63 Å: a typical distance found for analogous nonheme iron(IV)-oxo complexes in enzymes and model complexes (de Visser, 2006a,b,d, 2007; Godfrey et al., 2008; Quesne et al., 2016b; Cantú Reinhard and de Visser, 2017). The short peptide chain (Ser-Pro)_5_ we retained in the model as it has its proline residue tightly packed nearby the iron(IV)-oxo group.

**Figure 3:**
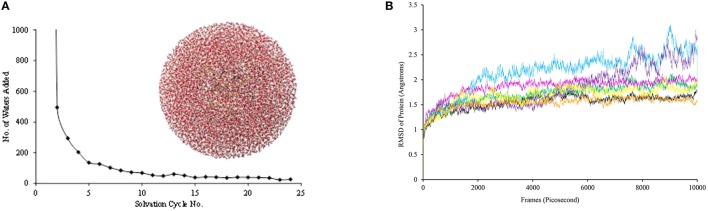
**(A)** Iterative solvation procedure for WT P4H. **(B)** MD simulations for WT and mutants of P4H. Colors represent WT (black), E127D (green), E127Q (pink), R161C (blue), R161D (purple), R161K (orange), and R161Q (yellow).

Subsequently, hydrogen atoms were added to the protein structure using the pdbtopqr program package assuming a pH = 7 (Dolinsky et al., 2007). Thus, all acid residues, i.e., Glu and Asp, were deprotonated whereas the basic residues, i.e., Arg and Lys, were protonated. The protonation state of each individual histidine residue was decided upon visual inspection of its local environment (donating/accepting hydrogen bonds) and we chose to assign all as singly protonated. Thereafter, the protein structure was solvated in a sphere with radius of 40 Å and energy minimized with the Charmm forcefield (Brooks et al., 1983). The solvation procedure was repeated a number of times until a situation was reached (Figure 3), whereby <20 water molecules were added to the chemical system. The saturated structure was then minimized without geometric constraints and heated to a temperature of 298 K. Finally, a full molecular dynamics (MD) simulation was run for 10 ns. The full set-up procedures were repeated for the mutant structures, whereby one amino acid was manually replaced. As follows from the MD simulations shown in Figure 3B, all converge well within 10ns. For each of the structures, we started QM/MM calculations using the snapshots taken after 5 ns (Sn_5ns_).

### QM/MM procedures

Density functional theory (DFT) methods were used to describe the QM region of the QM/MM calculation. In particular, we used the unrestricted hybrid density functional method B3LYP (Lee et al., 1988; Becke, 1993) in all cases as recent benchmark studies from our group showed this procedure to give rate constants in very good agreement with experiment (Cantú Reinhard et al., 2016a). In particular, QM methods with dispersion included were shown to underestimate free energies of activation considerably (Cantú Reinhard et al., 2016a). Furthermore, B3LYP was shown previously to predict regioselectivities and bifurcation pathways well as compared to experiment (Kumar et al., 2004; Barman et al., 2016; Brazzolotto et al., 2017). Also, DFT calculated free energies of activation were shown to match experimentally determined ones of biomimetic model complexes containing iron and manganese very well and reproduced Hammett trends (Vardhaman et al., 2011, 2013; Kumar et al., 2014; Yang et al., 2016). Here, DFT calculations were run in Turbomole (Ahlrichs et al., 1989), and the MM ones in DL-Poly with the Charmm forcefield (Smith and Forester, 1996). The ChemShell software package(Sherwood et al., 2003) interfaced Turbomole and DL-Poly and was used to obtain QM/MM energies and derivatives. The link-atom approach was used to describe atoms on the border between the QM and MM regions and essentially replaced a covalent bond with a C–H bond (Bakowies and Thiel, 1996). All calculations use electronic embedding of the charges of the MM region included into the QM Hamiltonian.

Geometry optimizations and reaction coordinate scans were done with an SV(P) basis set on all atoms: basis set BSI (Schafer et al., 1992). Reaction coordinate scans were run with one degree of freedom fixed and explored the potential energy surface between reactants, intermediates and products. The maxima of these scans were used as starting points for transition state searches. The energies of the stationary points were improved by running a single point calculation with an all-electron Wachters-type basis set on iron and def2-TZVP on the rest of the atoms: basis set BSII (Wachters, 1970).

### QM region

**Figure 4:**
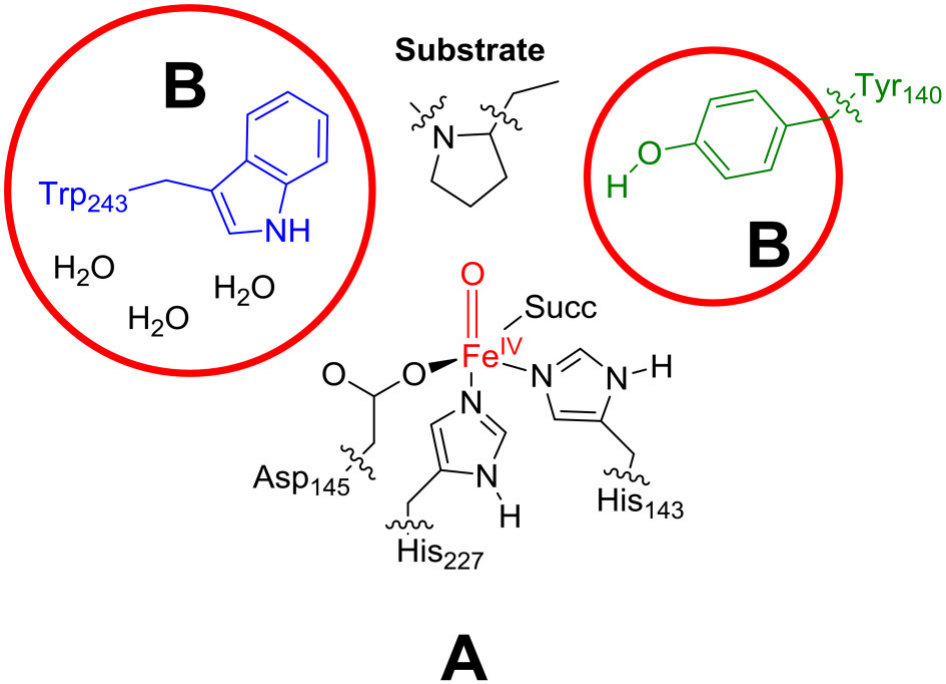
Description of QM region A and AB.

As the active site region of the protein contains many hydrogen bonding and π-stacking interactions we considered two QM regions: a minimal QM region A and an expanded QM region AB, see Figure 4. Thus, the minimal QM region A contains the iron(IV)-oxo group and its direct ligands (His_143_, His_227_, Asp_145_, and succinate) as well as the proline ring of the peptide substrate. The larger QM region AB was expanded with the indole ring of Trp_243_, the phenol group of Tyr_140_ and three water molecules.

## Results and discussion

### P4H WT structure

**Figure 5:**
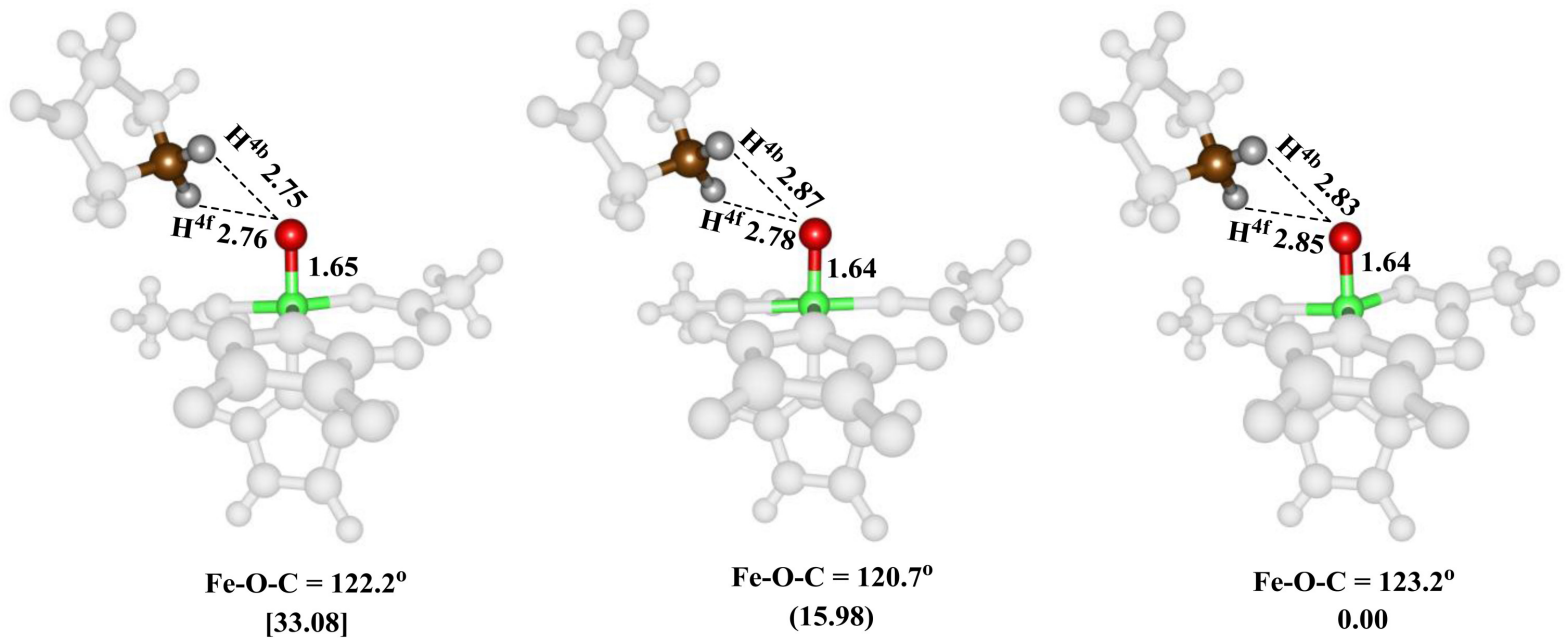
QM/MM optimized geometries of the low-lying spin states of the iron(IV)-oxo species of WT P4H for snapshot Sn_5ns_ and QM region A. From left-to-right, the data reported are for the singlet, triplet and quintet spin states. Bond lengths are in angstroms and relative energies in kcal mol^−1^.

The iron(IV)-oxo species (structure G in the catalytic cycle of Figure 2) was fully optimized with QM/MM methods using B3LYP/BSI and QM region A and AB, see Figure 5. In agreement with experimental EPR and Mössbauer spectroscopic studies (Hoffart et al., 2006) the quintet spin state is the ground state, while we located the triplet and singlet spin states higher in energy by 16.0 and 33.1 kcal mol^−1^, respectively. Geometrically, the iron(IV)-oxo species is bound to the protein with two Fe–N_His_ interactions of 2.06 and 2.08 Å, which is typical for metal-histidine interactions in proteins (de Visser et al., 2009). The carboxylate group of Asp_145_ binds as a monodentate ligand at a distance of 2.01 Å, whereas the succinate carboxylate group binds as a bidentate ligand with distances of 2.20 and 2.30 Å. Again, these distances match previous calculations on similar complexes nicely (Pratter et al., 2013). Substrate proline is not bound directly to the iron center but its transferring C–H^4b^ hydrogen atom is found at a distance of 2.86 Å from the oxo group and hence is positioned in the ideal orientation for oxidation. The triplet and singlet spin states give analogous ligand distances but are distinguished by their differences in iron(IV)-oxo bond length due to differences in molecular orbital occupation.

**Figure 6:**
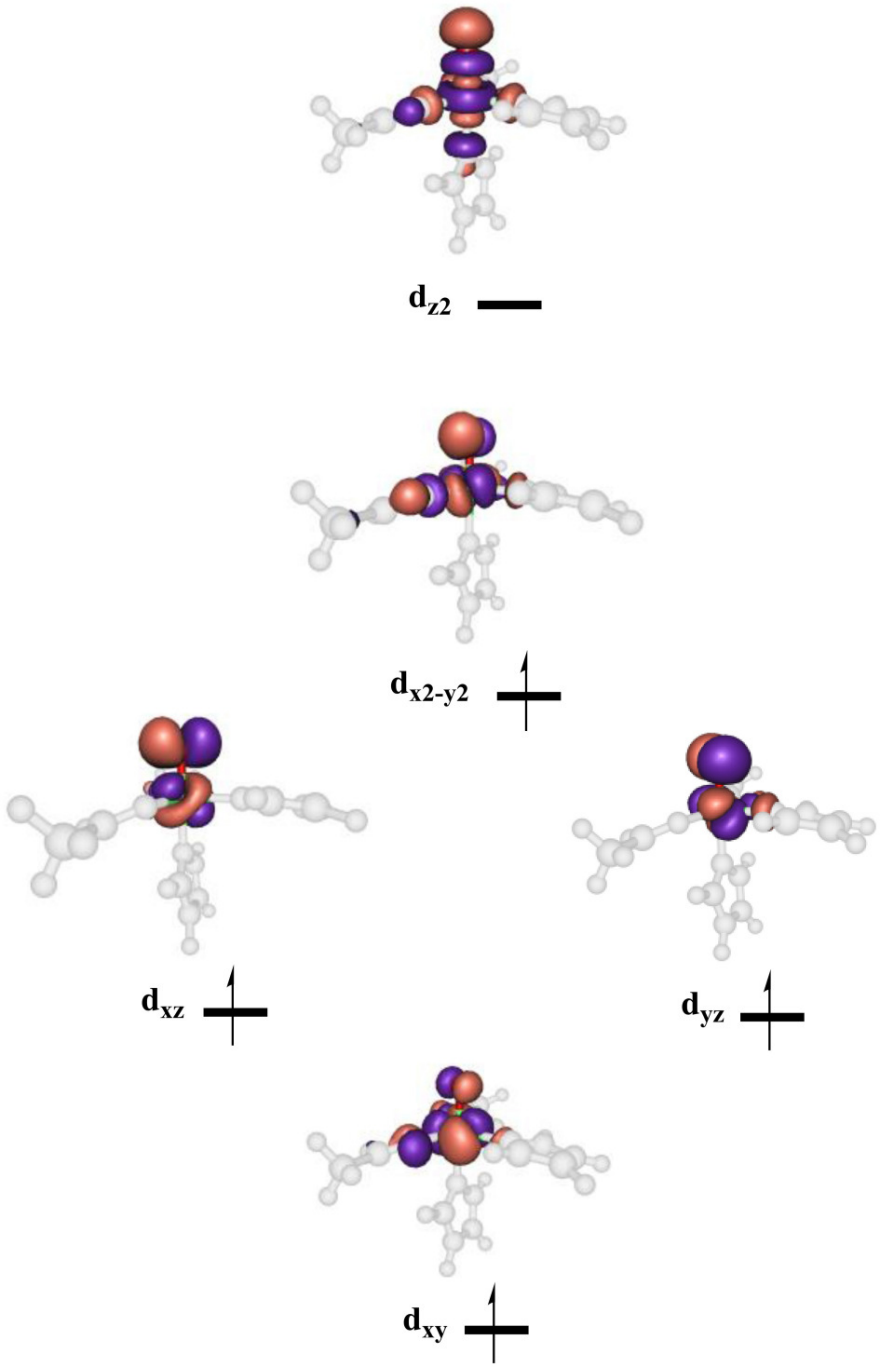
Molecular valence orbitals of the iron(IV)-oxo species of P4H.

High-lying occupied and low-lying virtual orbitals of the iron(IV)-oxo species are shown in Figure 6, which gives the molecular *z*-axis along the Fe–O bond. The lowest lying orbital is the π^*^_xy_ orbital that represents the interactions of the metal 3d_xy_ orbital with the equatorial ligands, namely His_143_, Asp_145_ and succinate (Succ). A bit higher in energy are the two orthogonal π^*^ orbitals along the Fe–O bond that correspond to the mixing of the 3d_xz_ on iron with 2p_x_ on oxygen, i.e., π^*^_xz_, and the 3d_yz_ on iron with the 2p_y_ on oxygen, i.e., π^*^_yz_. Higher in energy still are two σ-type orbital interactions. The first one along the *z*-axis for the mixing of the metal 3d_z2_ with a 2p_z_ on oxygen: $\sigma_{\text{z}2}^{*}$. The second one is located in the *xy*-plane and results from the interaction of the 3d_x2−y2_ orbital on iron with orbitals on the ligands: σ^*^_x2−y2_. The quintet spin state has orbital occupation ${\pi^{*}}_{\text{xy}}^{1}$ ${\pi^{*}}_{\text{xz}}^{1}$ ${\pi^{*}}_{\text{yz}}^{1}$ ${\sigma^{*}}_{\text{x}2-\text{y}2}^{1}$ ${\sigma^{*}}_{\text{z}2}^{0}$, while the triplet spin state has configuration ${\pi^{*}}_{\text{xy}}^{2}$ ${\pi^{*}}_{\text{xz}}^{1}$ ${\pi^{*}}_{\text{yz}}^{1}$ ${\sigma^{*}}_{\text{x}2-\text{y}2}^{0}$ ${\sigma^{*}}_{\text{z}2}^{0}$. Generally, enzymatic iron(IV)-oxo species tend to have a quintet spin ground state (Latifi et al., 2009), while most synthetic biomimetic models have a triplet spin ground state (de Visser et al., 2012). Computational studies showed that this is the result of differences in coordination system, where biomimetic models are often in octahedral coordination, whereas enzymatic structures have the iron(IV)-oxo in pentacoordination (Latifi et al., 2013).

### P4H hydroxylation mechanisms

**Figure 7:**
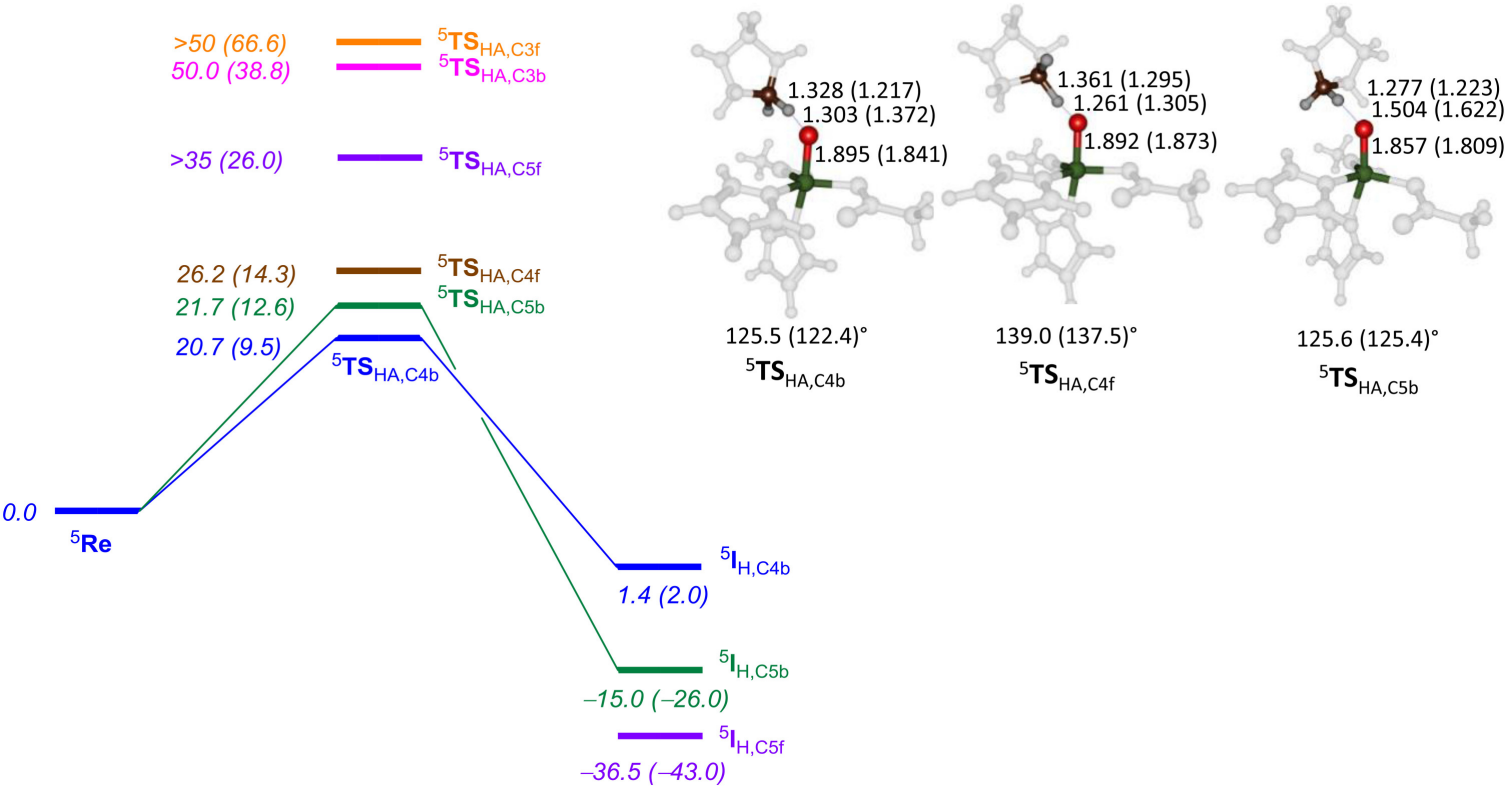
QM/MM calculated hydrogen atom abstraction barriers for P4H WT with energies in kcal mol^−1^ as obtained with QM region AB (A). Optimized geometries of the lowest energy hydrogen atom abstraction transition states are given with bond lengths in angstroms and angles in degrees.

Subsequently, we investigated proline hydroxylation at the C^3^, C^4^ and C^5^ position of P4H and considered both hydrogen atoms at each of these positions. All reactions were found to be stepwise with an initial hydrogen atom abstraction via transition state **TS**_HA_ to form a radical intermediate **I**_H_. A radical rebound step via **TS**_reb_ then produced the alcohol product complexes **P**. In all cases the rebound step was small and the hydrogen atom abstraction was rate-determining. The QM/MM calculated energy landscapes are given in Figure 7 for hydrogen atom abstraction from C^4f^, C^4b^, C^5f^, C^5b^, C^3f^, and C^3b^. The lowest energy barrier height is the one for C^4b^ and after rebound will give the *R*-4-hydroxyproline product complex, which is the experimentally determined stereo- and regioselective product found. Although the difference in relative energies is small, the calculations predict the correct regio- and stereoselectivity. Slightly higher in energy (1 kcal mol^−1^ using basis set BSII and 2.9 kcal mol^−1^ using basis set BSI) we find the pathway for hydrogen atom abstraction from C^5b^, the thermodynamically more favorable pathway. Indeed the radical intermediate for C^5b^ is much lower in energy than the one for C^4b^ in agreement with the thermodynamic predictions. About 5.5 kcal mol^−1^ higher in energy than ^5^**TS**_HA,C4b_ is the barrier ^5^**TS**_HA,C4f_, which implies that there are strong energetic differences between hydrogen atom abstraction of the two hydrogen atoms on carbon center C^4^. The three barriers for hydrogen atom abstraction from C^5f^, C^3f^, and C^3b^ are all well higher in energy than ^5^**TS**_HA,C4b_ by at least 15 kcal mol^−1^ and hence will play little role of importance. As the singlet and triplet spin states were already considerably higher in energy at the reactant stage, they remain well higher in the hydrogen atom abstraction transition states as well. The triplet and singlet spin barriers for abstracting the C^4b^ hydrogen atoms are 34.0 and 40.2 kcal mol^−1^ at UB3LYP/BSI in QM/MM. As such the reactivity takes place on a single spin state only, the quintet spin state, and other spin states play no role in the rate-determining pathway.

The optimized geometries of ^5^**TS**_HA,C4b_, ^5^**TS**_HA,C4f_, and ^5^**TS**_HA,C5b_ (right-hand-side of Figure 7) give insight into their energetic ordering and relative energies. Thus, in ^5^**TS**_HA,C4b_ the transferring hydrogen atom is almost midway in between donor and acceptor atom and the Fe–O–C^4b^ angle is about 125°. In ^5^**TS**_HA,C4f_, by contrast, the substrate is oriented along a much larger angle of 139.0°. The ^5^**TS**_HA,C5b_ structure, on the other hand, has the transferring hydrogen atom at a relatively large distance from the accepting oxygen atom and hence is destabilized considerably.

### P4H mutations of Arg_161_

To find out the effect of substrate positioning and catalytic turnover of active site mutations, we investigated several P4H models, where amino acids were replaced. Thus, in our previous studies (Timmins et al., 2017) we implicated an important role of Arg_161_ and Glu_127_ through hydrogen bonding interactions. In this section we will look into the structure and catalytic activity of P4H mutants with Arg_161_ replaced by either Asp, Gln, or Lys. These changes could be dramatic as the Arg161Asp (R161D) mutation will replace a positively charged residue with a negatively one. Similarly, the Arg161Gln (R161Q) mutation changes a cationic residue into a neutral one.

**Figure 8:**
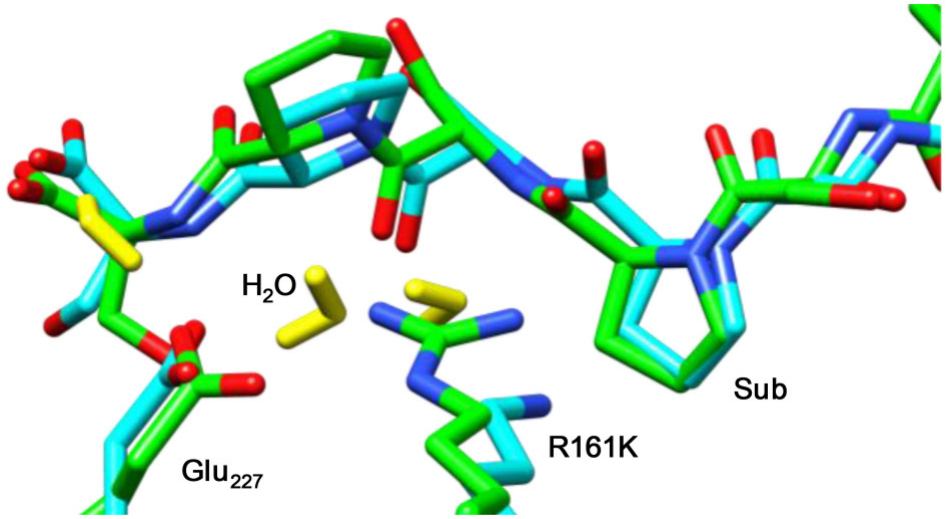
Overlay of the iron(IV)-oxo structures of WT P4H and R161K mutants as optimized with QM/MM. Bond lengths are in angstroms.

Figure 8 displays an overlay of the structures of the iron(IV)-oxo species for WT and R161K mutation after a full QM/MM geometry optimization, where the positively charged Arg residue is replaced by the positively charged Lys amino acid. As can be seen the mutation displaces the salt bridge between Arg_161_ and Glu_127_, which bends outward. The space provided by the Glu_127_ migration is filled up with extra water molecules. However, the removal of the strong hydrogen bonding interactions of the Arg_161_-Glu_127_ couple toward the substrate has a major effect on the stability of the substrate and its positioning. Thus, substrate is lesser tight bound in the R161K mutant than in WT and hence its regio- and stereoselective substrate activation may be affected.

**Figure 9:**
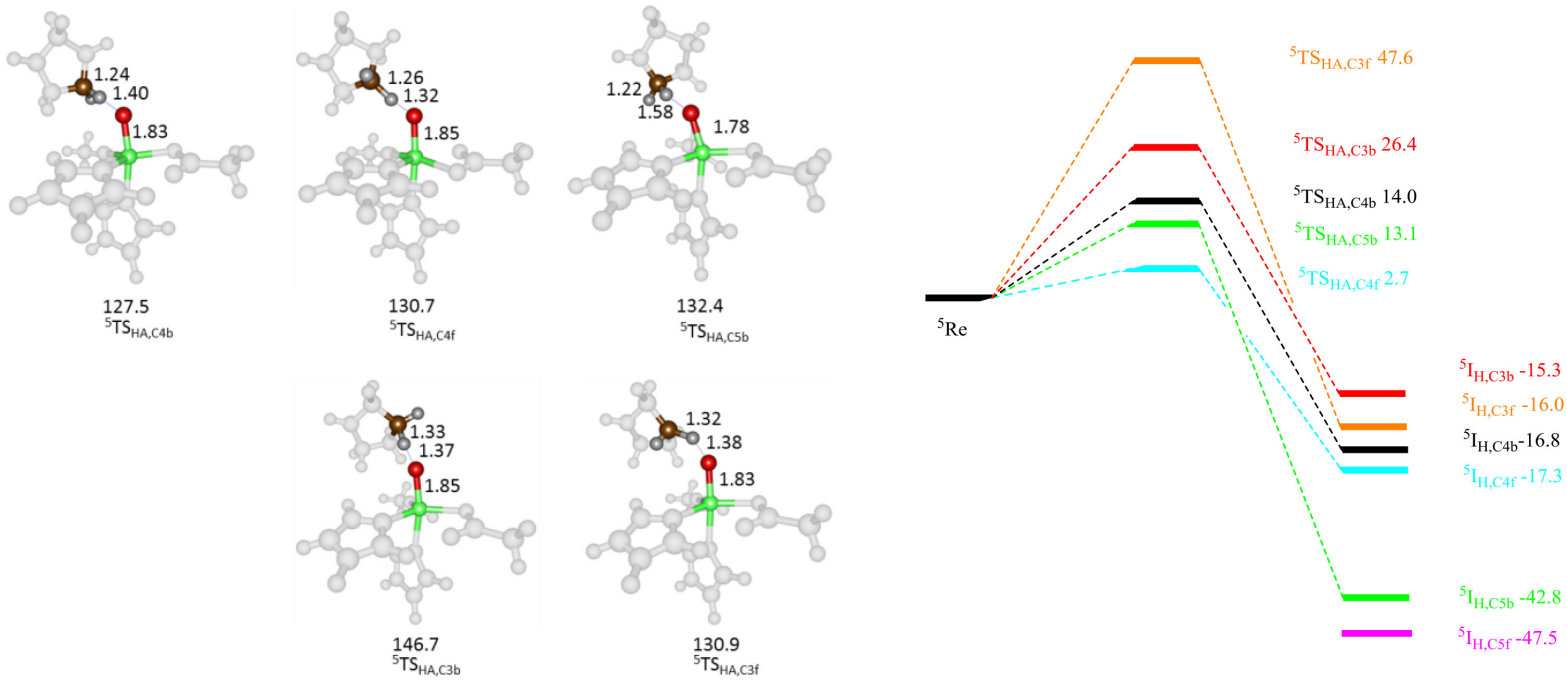
QM/MM calculated hydrogen atom abstraction barriers for P4H R161K with energies in kcal mol^−1^ as obtained with QM region A. Optimized geometries of the lowest energy hydrogen atom abstraction transition states are given with bond lengths in angstroms and angles in degrees.

Subsequently, we studied the hydrogen atom abstraction mechanisms of the R161K mutant from the C^3^, C^4^, and C^5^ positions of proline for the back and front protons. Figure 9 displays relative energies and optimized geometries of selected hydrogen atom abstraction transition states for the R161K mutant. The hydrogen atom abstraction barrier from the C^4b^ position (^5^**TS**_R161K,C4b_) has an energy of 14.0 kcal mol^−1^ (UB3LYP/BSI), which is almost identical to the one observed for WT of 14.3 kcal mol^−1^. Indeed, the optimized geometries are very similar: C–H and O–H distances are found of 1.24 and 1.40 Å for R161K, whereas they are 1.22 and 1.37 Å, respectively, for WT (Figure 7 above). However, a much lower transition state is found for activation of the C^4f^ position of only 2.7 kcal mol^−1^. Therefore, the R161K mutation will not affect the catalytic performance of the enzyme: It should react faster than WT, but will give a reversal of stereochemistry and predominantly produce the *S*-4-hydroxyproline product instead.

Comparison of ^5^**TS**_C4b_ transition state structures in R161K to WT shows that the positions of the active site components change little, however, the Fe-O-C^4^ angle and N_His_-Fe-O-C^4^ dihedral angle are different, namely 137.5° and −73.4° for WT and 130.7° and −67.5° for R161K, respectively. Therefore, this mutation allows for the orientation of the substrate relative to the iron(IV)-oxo to change in such a way that the C^4f^ position is now accessible to it. Hydrogen atom abstraction from the C^3^ position is seen to be lowered as compared to WT but is significantly higher in energy than the barrier ^5^**TS**_R161K,C4f_. The barrier for C^5b^ hydrogen atom abstraction is similar to that for C^4b^ but now slightly lower in energy.

**Figure 10:**
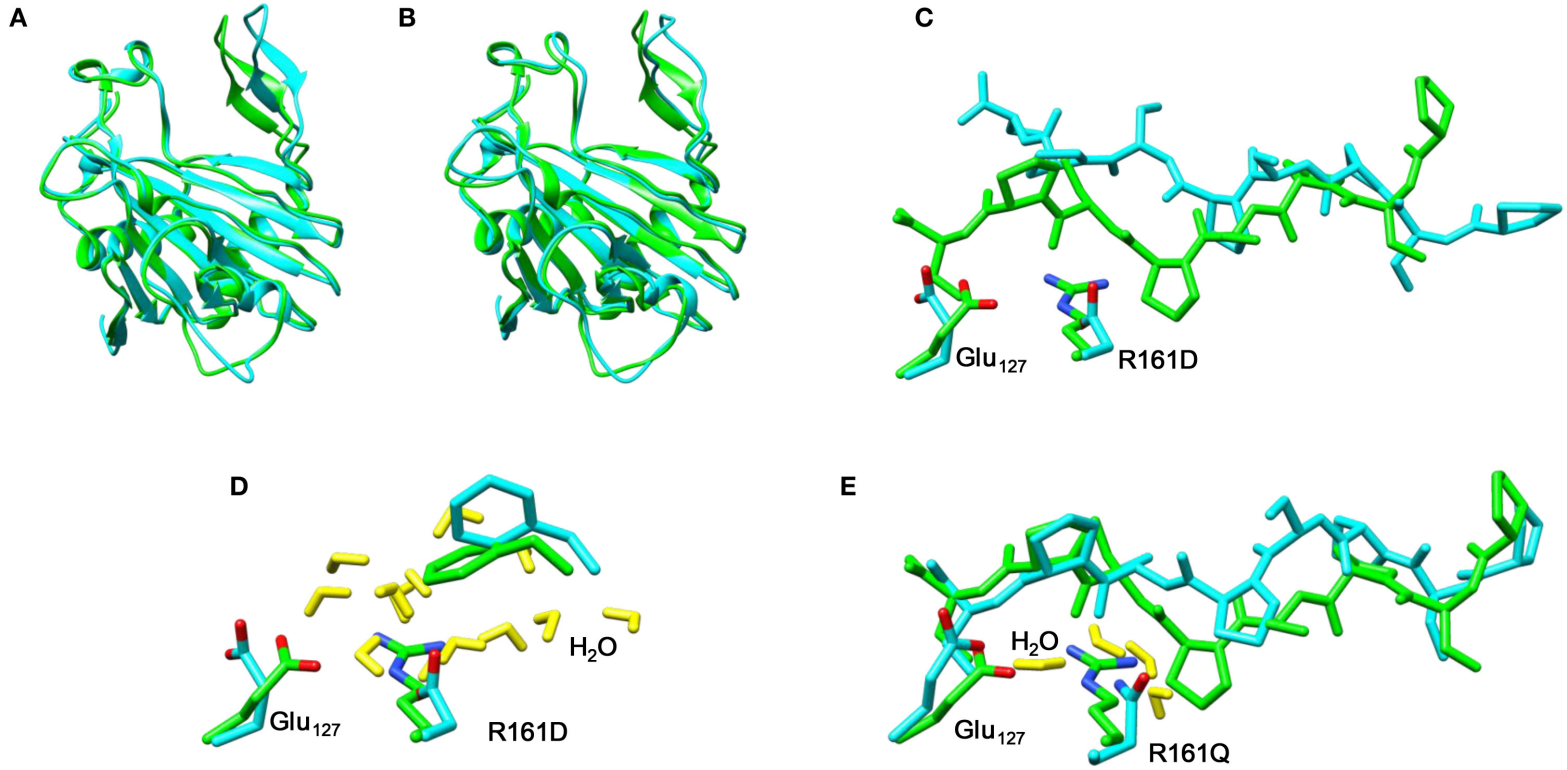
Overlay of the iron(IV)-oxo structures of WT P4H (in green) and Arg_161_ mutants (in blue) as optimized with QM/MM. **(A)** WT vs R161D **(B)** WT vs R161Q **(C)** WT vs R161D **(D)** WT vs R161D **(E)** WT vs R161Q.

Thereafter, we studied the R161Q and R161D mutants and Figure 10 gives structures of the iron(IV)-oxo species as compared to WT. As can be seen both mutations have a dramatic effect on substrate binding and positioning as a result of changes in the hydrogen bonding network between Glu_127_, R161D and surrounding residues located in the β_II_-β_III_ and β_3_-β_4_ loops. We then attempted to abstract hydrogen atoms from proline by the R161Q and R161D mutants. Table 1 gives data with calculated barrier heights for several hydrogen atoms of proline. None of these barrier heights, however, is low enough in energy to make them accessible at room temperature. Therefore, the R161Q and R161D mutants will be catalytically inactive. As a result, the Arg_161_ residue has a critical function in P4H enzymes in positioning the substrate in the correct orientation. This is done in conjunction with the Glu_127_ residue that hydrogen bonds the protein loop of the substrate and makes sure it can approach the iron(IV)-oxo species.

**Table 1:** Calculated barrier heights (kcal mol^−1^) for several hydrogen atom transfers from the substrate proline residue to the iron(IV)-oxo oxidant in prolyl-4-hydroxylase.

	**^5^TS_HA,C4b_**	**^5^TS_HA,C4f_**	**^5^TS_HA,C5b_**	**^5^TS_HA,C5f_**	**^5^TS_HA,C3b_**	**^5^TS_HA,C3f_**
WT	9.8	14.8	12.7	27.6	38.4	66.6
R161D	60.4	60.8	45.4	68.4	85.3	73.8
R161Q	46.1	48.3	N/A	N/A	N/A	62.8

### P4H mutations of Glu_127_

**Figure 11:**
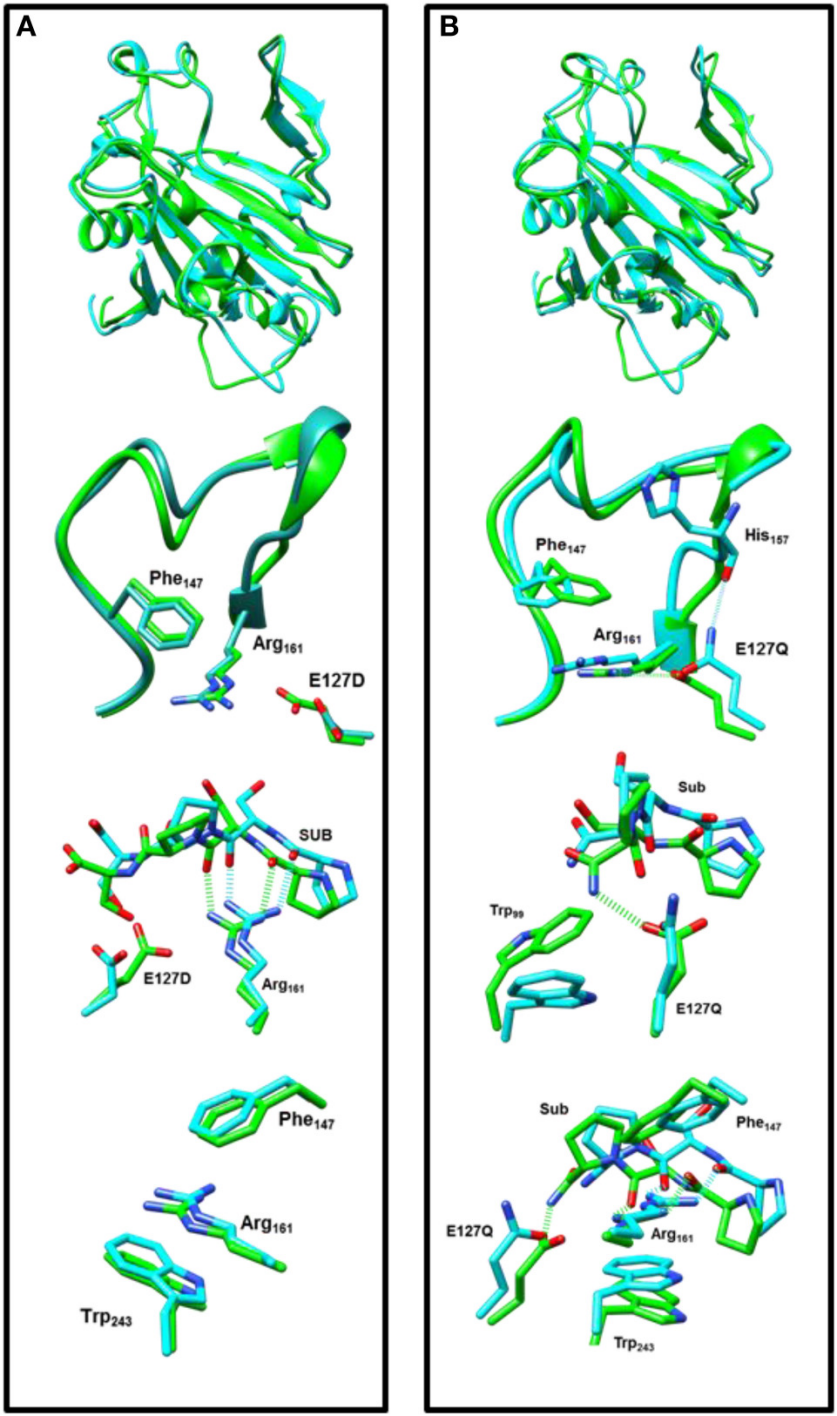
Overlay of the iron(IV)-oxo structures of WT P4H (in green) and Glu_127_ mutants (in cyan) as optimized with QM/MM. **(A)** WT vs. E127D and **(B)** WT vs E127Q.

In a final set of calculations we investigated P4H mutants where Glu_127_ is replaced by either Asp or Gln. Figure 11 displays the QM/MM optimized iron(IV)-oxo species of WT version E127D and E127Q mutants. In E127D the hydrogen bond between Asp_127_ and Arg_161_ is broken and as a result as Asp_127_ swings out, to subsequently effecting the positions of surrounding residues and the substrate. Interestingly, the hydrogen bond between Arg_161_ and the substrate is maintained; however, the position of this residue corresponds to the change in the substrate position. It suggests that the function of Glu_127_ is to anchor Arg_161_ in a fixed position, which is essential for proper substrate positioning. In E127Q, there are notable changes around the active site with, e.g., Tyr_140_ rotating out of its WT position breaking its hydrogen bond to the iron(IV)-oxo which has previously been shown to be important for correct substrate positioning and release. Previous research (Koski et al., 2009) has suggested that any disturbance of this “conformational switch” would result in the inactivation of the enzyme as shown by its mutation to alanine in experiment (Koski et al., 2009). The position of Trp_243_ is also altered affecting substrate positioning even more, and consequently the E127Q mutation is likely to lead to an inactive form of the enzyme. To test the hypothesis, we explored hydrogen atom abstraction from various positions of proline residue which are given in Table 2.

**Table 2:** Calculated barrier heights (kcal mol^−1^) for several hydrogen atom transfers from the substrate proline residue to the iron(IV)-oxo oxidant in prolyl-4-hydroxylase.

	**^5^TS_HA,C4b_**	**^5^TS_HA,C4f_**	**^5^TS_HA,C5b_**	**^5^TS_HA,C5f_**	**^5^TS_HA,C3b_**	**^5^TS_HA,C3f_**
WT	9.8	14.8	12.7	27.6	38.4	66.6
E127D	11.6	20.8	12.5	43.1	30.0	44.1
E127Q	25.0	N/A	20.1	41.8	42.5	55.6

In E127D, no longer is hydrogen atom abstraction (HAT) from C^4b^ the favored pathway, but now, HAT from C^5b^ is within 1 kcal mol^−1^ and hence the two pathways are competitive. Considering the energies of the intermediate radical species in the mutant, it becomes clear that the C^5b^ structure (−31.8 kcal mol^−1^) product will be the major product over the C^4b^ (−2.9 kcal mol^−1^), therefore the E127D mutation leads to a change in the regioselectivity of the reaction. This is similar to what has been seen in previous DFT calculations which showed that if given the choice HAT from the C^5^ position will always be favored over the C^4^ position as the BDE at the former position is weaker compared to the latter (Karamzadeh et al., 2010). Comparison of the ^5^TS_C5b_ transition state structures in WT and E127D reveal similar geometric parameters, however, the Fe-O-C^5^ angle and N_His_-Fe-O-C^5^ dihedral angle are different, namely 125.4° and −77.8° for WT and 132.1° and −88.2° for E127D, respectively. As such, the orientation of the substrate relative to the iron(IV)-oxo has changed, allowing the substrate in E127D to adopt a more favorable position for HAT from the C^5b^ position as compared to WT. In E127Q, HAT from the C^5b^ position is favored compared to that from C^4b^, as shown also for E127D. However, as the transition state barrier is 20.1 kcal mol^−1^ for HAT from the C^4b^ position, the mutant will result in much slower reactivity as compared to WT.

In conclusion, previous QM/MM studies on P4H have elucidated the reasoning behind its observed regioselectivity and stereoselectivity in the WT through various mutations to the protein. The results of that study highlighted the role of Glu_127_ and Arg_161_ in substrate positioning and suggested how mutating those residues could alter the regioselectivity and stereoselectivity with minimal influence on the enzymes stability and catalytic ability, an important consideration for future biotechnological applications. This study has revealed that mutations E127D and R161K are possible mutation candidates which will result in a change in the regioselelctivity and stereoselectivity. Additionally, the work highlights the importance in conserving the charge in the substrate binding residues of the enzyme around the substrate cavity to ensure interactions between the substrate and protein are maintained when an amino acid is mutated.

## Conclusion

Here we describe a detailed computational study into the activity of prolyl-4-hydroxylase enzymes and several Glu_127_ and Arg_161_ mutants. In particular, a comprehensive QM/MM study is presented, whereby we investigated hydrogen atom abstraction channels of each pair of hydrogen atoms bound to C^3^, C^4^, and C^5^ of the proline residue of the substrate. Studies on WT predict the experimentally observed product distributions and give regio- and enantioselective *R*-4-hydroxyproline as a product. Analysis of the structure and electronic configurations show the regioselectivity to be guided by substrate positioning and hydrogen bonding interactions. Mutations of Glu_127_ and Arg_161_ have major effects and lead to inactivity of the protein in several cases, in particular, when an anionic residue is replaced by a cationic one. Only in the case of the E127D mutant significant activity remains although competitive C^4b^ and C^5b^ hydroxylation is predicted.

## Author contributions

AT and SdV designed and developed the project. AT performed the calculations. AT and SdV wrote the paper.

### Conflict of interest statement

The authors declare that the research was conducted in the absence of any commercial or financial relationships that could be construed as a potential conflict of interest. The reviewer, TM, and handling Editor declared their shared affiliation.
